# Differential Clinical Outcome of Dengue Infection among Patients with and without HIV Infection: A Matched Case–Control Study

**DOI:** 10.4269/ajtmh.15-0031

**Published:** 2015-06-03

**Authors:** Junxiong Pang, Tun-Linn Thein, David C. Lye, Yee-Sin Leo

**Affiliations:** Communicable Diseases Center, Institute of Infectious Diseases and Epidemiology, Tan Tock Seng Hospital, Singapore; Saw Swee Hock School of Public Health, National University of Singapore, Singapore; Department of Medicine, Yong Loo Lin School of Medicine, National University of Singapore, Singapore; Lee Kong Chian School of Medicine, National Technological University, Singapore

## Abstract

Clinical characteristics and outcome among dengue patients with and without human immunodeficiency virus (HIV) infection remain elusive. A total of 10 dengue virus (DENV)–HIV Chinese patients were compared with 40 Chinese dengue patients without HIV, who were matched for age, gender, type of care received, methods, and year of dengue diagnosis from 2005 to 2008. Univariate and multivariate conditional logistics regression were applied. DENV-HIV patients were significantly associated with the World Health Organization (WHO) 2009 severe dengue (conditional odds ratio [COR] = 5.72; 95% confidence interval [CI] = 1.01–32.64) but not with the WHO 1997 dengue hemorrhagic fever/dengue shock syndrome (COR = 0.40; 95% CI = 0.09–1.71). This is mainly due to severe plasma leakage and the lack of hemorrhagic manifestations. Hospitalization duration was longer for DENV-HIV patients (10.5 days; interquartile range [IQR] = 5.5–26.3 days) compared with dengue patients (5 days; IQR = 4–6 days). There were no significant differences in presentation of clinical warning signs and symptoms at admission and during hospitalization, except for rash (adjusted COR [ACOR] = 0.06; 95% CI = 0.03–0.92). DENV-HIV patients were associated with higher pulse rate (ACOR = 1.13; 95% CI = 1.02–1.25), eosinophils proportion (ACOR = 3.07; 95% CI = 1.12–8.41) and lower hematocrit level (ACOR = 0.79; 95% CI = 0.64–0.98) compared with dengue patients. Even though DENV-HIV patients may present similarly to dengue patients, they may be more likely to have severe dengue outcome. Hence, close monitoring of DENV-HIV patients is highly recommended as part of dengue clinical care and management.

## Introduction

Dengue is caused by four closely related dengue virus strains 1–4 (DENV 1–4), which are positive-sense, single-stranded RNA viruses that belongs to the family Flaviviridae. Dengue is endemic in the tropical and subtropical areas of the world, where human immunodeficiency virus (HIV), a human retrovirus of the family Retroviridae that causes the acquired immunodeficiency syndrome (AIDS) pandemic, is also likely to be prevalent.^1^–^3^ Despite the overlapping epidemiology, knowledge on the differential clinical manifestations and disease severity between DENV-HIV coinfected and DENV patients is limited. Only a handful of case reports^4^–^6^ and case series were published thus far.^7^

Previous published studies suggested that patients who had DENV-HIV coinfection were likely to have non-severe clinical illness. It was observed that there was no increase in dengue hemorrhagic fever/dengue shock syndrome (DHF/DSS) in individuals with coinfection. No acceleration of HIV disease was also reported in two male patients coinfected with DENV 3 and HIV in Cuba.^6^ In addition, Singapore reported five DENV-HIV patients with no severe outcomes, and four out of the five were males.^7^ Furthermore, a male patient in Sao Luis, Brazil, who was coinfected, did not have any life-threatening complications although fulfilling the criteria of DHF.^4^ Another study suggested that there might be a transient suppression of HIV-1 replication during an acute DENV infection.^5^ Further studies suggested that this transient suppression was likely due to the role of DENV NS5 protein downregulating HIV co-receptor (CXCR4) expression and the increased production of stromal cell–derived factor 1 (SDF-1), a chemokine ligand for CXCR4.^8^,^9^ However, these small series and anecdotal case reports cannot fully conclude whether DENV-HIV coinfected patients are not at increased risk of severe disease.

With the increasing trend of dengue and HIV infection in Asia,^1^–^3^,^10^,^11^ it is timely to achieve a better understanding of the DENV-HIV coinfection through systematic analysis. We performed a review of a large cohort database to assess disease severity and differences in clinical and laboratory characteristics among DENV patients with and without HIV at first presentation to our institution and during hospitalization.

## Methods

A matched case–control study was conducted using anonymized data collected from all adult dengue patients admitted from January 1, 2005 to December 31, 2008 to the Communicable Disease Center (CDC) at Tan Tock Seng Hospital (TTSH). This was the largest hospital in Singapore for the treatment of dengue patients where they were managed using a standardized dengue care-path as reported in other study.^12^ In addition, the CDC was the National Referral Center for HIV patients, which managed close to 90% of HIV patients in Singapore.^13^ All HIV patients were confirmed by positive HIV enzyme-linked immunosorbent assay (ELISA) and Western blot at our national reference laboratory.

During the study period from 2005 to 2008, Singapore experienced two predominant serotypes at different period; serotype 1 was detected in 75–100% of dengue samples during the epidemics in the year 2005–2006 and dengue serotype 2 was detected in up to 91% dengue samples during the epidemic in the year 2007 and 2008.^14^ Each DENV-HIV coinfected patient was randomly matched to four DENV patients without known HIV infection by age, gender, laboratory diagnostic methods for dengue, year of dengue diagnosis, and the type of care at provision site (mainly outpatient or inpatient care) as controls. Singapore is a multiracial country having Chinese as the majority. Since only Chinese patients were found to be DENV-HIV coinfected, the controls were matched to only Chinese DENV patients. All clinically suspected patients were tested with dengue polymerase chain reaction (PCR) assay, dengue immunoglobulin-M (IgM) and IgG. DENV patients either had positive PCR assay or positive IgM or IgG (Dengue Duo IgM and IgG Rapid Strip; Panbio Diagnostic, Queensland, Australia), and fulfilling either the WHO 1997^15^ or 2009^16^ probable dengue criteria. As the main aim of the study was identifying differential dengue presentation and severity due to the coinfection at first presentation and final outcome, instead of the implications of the viral kinetics and interactions between the two viruses, it should not matter to a great extent on whether the dengue patients were diagnosed with PCR, IgM or IgG positive assay. Furthermore, these patients have had to fulfill the WHO probable dengue criteria.

Data at first presentation in CDC and during the course of hospitalization was obtained from medical records. These included demographic, epidemiological, comorbidities, and clinical and laboratory results. Disease severity was determined at first presentation and at recovery from dengue, according to the WHO 1997^15^ and 2009 classifications,^16^ with severe plasma leakage and clinical fluid accumulation as defined in previous published study.^12^ The duration of disease progression to DHF/DSS and severe dengue post presentation was assessed only for patients who were classified as dengue fever (DF) and “probable dengue with/without warning signs” at first clinical presentation, respectively. The number of days post presentation (DPP) was used to define the period since the first presentation in hospital. The number of days post fever (DPF) onset was used to define the period since the day of fever onset.

### Statistical methods.

Univariate and multivariate conditional logistic regression were performed to assess the association between the variables of interests and DENV-HIV coinfection. Conditional logistic regression was used to account for the set of matching factors selected in this study as described above. Matching was performed on the year of dengue diagnosis to control for potential confounding by differing circulating predominant serotype. Confounding effect was further minimized by performing multivariate conditional logistic regression adjusting for the DPF at first presentation. The laboratory variables were analyzed in the continuous format to maximize the data available and to minimize reporting bias that might occur when the variables were categorized into the expected clinically normal or hypothetical range. All statistical analyses were performed using Stata 10.0 (STATA Corp., College Station, TX). All tests were conducted at the 5% level of significance, with conditional odds ratio (COR) and/or adjusted COR (ACOR), *P* value, and corresponding 95% CI reported where applicable.

### Ethics statement.

This study was approved by Domain Specific Review Board, National Healthcare Group, Singapore (DSRB-E/08/567) with waiver of informed consent as this was a retrospective study and the data were analyzed anonymously.

## Results

A total of 8,123 records of dengue patients were available between January 2005 and December 2008. There were a total of 10 DENV-HIV coinfected cases identified (0.12%) (Table 1), which was comparable to the national HIV prevalence rate of 1,155 per million population (0.12%).^17^ The median age of these DENV-HIV cases was 47 years (interquartile range [IQR] = 39–58 years of age) (Supplemental Table 1). There were 9 (90%) male, one with diabetes, one with hypertension, and one with asthma (Table 1 and Supplemental Table 1). Among the cases, there were 30% positive for dengue PCR assay and 70% were serology positive (Table 1 and Supplemental Table 1). The median DPF at presentation for dengue was 3 days (IQR = 2–4.8 days) and 4 days (IQR = 4–5.3 days) for cases and controls, respectively (Table 2). DENV-HIV patients were significantly associated with earlier presentation at CDC (COR = 0.47; 95% CI = 0.24–0.94). Diabetes mellitus and renal disorder were more common among cases (10% and 20%, respectively) compared with controls (7.5% and 5%, respectively), whereas hypertension was less common among cases (10%) compared with controls (20%). None of these comorbidities were significantly associated with DENV-HIV cases (diabetes COR = 1.33; 95% CI = 0.14–12.82; hypertension COR = 0.43; 95% CI = 0.05–4.05; renal disorder COR = 4; 95% CI = 0.56–28.40) (Supplemental Table 1).

### Dengue severity and clinical outcomes.

Among the DENV-HIV cases, seven had fulfilled the criteria of AIDS and eight were on antiretroviral therapy (Table 1). The median period from HIV diagnosis to DENV diagnosis was 36.5 months (IQR = 16–52.75 months) (Supplemental Table 1). The median CD4 counts 6 months before and after dengue diagnosis were 123 cells/mm^3^ (IQR = 79–303 cells/mm^3^) and 144 cells/mm^3^ (IQR = 74.5–251 cells/mm^3^), with median lowest CD4 count of 45 cells/mm^3^ (IQR = 37–75.5 cells/mm^3^) (Supplemental Table 1). Of the seven HIV cases who had viral load assessment prior to dengue diagnosis, the median HIV viral load was 50 copies/mL (IQR = 50–251 copies/mL) (Supplemental Table 1).

Applying the WHO 1997 dengue classification at first presentation to CDC, none of the cases were classified as DHF/DSS compared with 30% controls (Table 2). For final clinical outcome, there were 30% cases and 52.5% controls being classified as DHF/DSS. In contrast, when applying the WHO 2009 dengue classification, 10% cases and 15% controls were classified as severe dengue at first presentation to CDC. For final clinical outcome, DENV-HIV patients were less likely to fulfill DHF/DSS (COR = 0.40; 95% CI = 0.09–1.71) criteria, but more likely to fulfill severe dengue (COR = 5.72; 95% CI = 1.01–32.64) criteria (Table 2). Although the severe dengue outcome based on the WHO 2009 dengue classification was barely statistically significant with *P* value of 0.049 (95% CI = 1.01–32.64), it is likely to have a large clinical significance with about six times higher risk of having severe dengue among DENV-HIV patients compared with DENV patients. This large clinical significance, to a major extent, should already justify its importance and relevance for clinical triage purpose. Five out of ten (50%) DENV-HIV patients fulfilled the WHO 2009 severe dengue criteria. Four (80%) out of five had severe plasma leakage. Of these four, one also had severe bleeding (Patient 3) and another one had severe organ involvement (Patient 6). The last severe dengue case (Patient 5) had severe bleeding, but no severe plasma leakage (Table 1).

The median days from first presentation to progression to DHF and severe dengue among cases was 6 days (range: 2–7 days) and 2 days (range: 2–3 days), respectively (Table 2). Among the controls, the median days to DHF/DSS and severe dengue was 3 days (range: 2–4 days) and 2.5 days (range: 2–3 days), respectively (Table 2). The median length of stay (LOS) in hospital was 10.5 days (IQR = 5.5–26.3 days) and 5 days (IQR = 4–6 days) for cases and controls, respectively (Table 2).

### Differential clinical characteristics of DENV-HIV patients.

At first presentation, DENV-HIV patients were significantly associated with higher pulse rate (ACOR = 1.13; 95% CI = 1.02–1.25) (Table 3), but the warning signs based on the WHO 2009 classification were not observed to be significantly different between DENV-HIV and DENV patients (Supplemental Table 2). In addition, other signs and symptoms such as hemorrhagic manifestation, rash, leucopenia, nausea/vomiting, ache and pains, thrombocytopenia and tachycardia were not observed to be significantly different (Supplemental Table 2). Even though, 5% DENV patients had severe bleeding and severe organ involvement compared with none among the DENV-HIV patients, statistical significance could not be assessed (Supplemental Table 2).

During hospitalization, DENV-HIV patients had lower risk of hemorrhagic manifestation (COR = 0.16; 95% CI = 0.03–0.81), rash (ACOR = 0.16; 95% CI = 0.03–0.92), and higher pulse rate (ACOR = 1.11; 95% CI = 1.01–1.22) (Table 3). Moreover, clinical fluid accumulation (ACOR = 3.18; 95% CI = 0.21–48.8), hepatomegaly (ACOR = 13.3; 95% CI = 0.54–334), and severe organ involvement (ACOR = 4.03; 95% CI = 0.002–83,615) were associated with DENV-HIV patients, but the differences did not reach statistical significance (Table 3 and Supplemental Table 3.

### Differential laboratory characteristics of DENV-HIV patients.

At first presentation, DENV-HIV patients were significantly associated with higher eosinophils proportions (ACOR = 3.07; 95% CI = 1.12–8.41) and lower hematocrit (ACOR = 0.79; 95% CI = 0.64–0.98) (Table 4). Similarly, during hospitalization, higher eosinophils proportion (ACOR = 1.90; 95% CI = 1.17–3.09), lower hematocrit level (ACOR = 0.74; 95% CI = 0.56–0.99), and lower serum potassium level (ACOR = 0.001; 95% CI = 0.001–0.81) were significantly associated with DENV-HIV patients (Table 4, Supplemental Table 4 and 5).

## Discussion

Dengue and HIV are both highly prevalent infections in Asia, resulting in substantial public health burden.^1^–^3^ However, the severity and characteristics of dengue and HIV (DENV-HIV) coinfection and the reciprocal impact on disease progression remain elusive because of lack of systematic case–control analysis.^4^,^5^,^7^,^18^ Thus far, case series had suggested that patients with DENV-HIV coinfection had non-severe dengue outcome and showed no signs of accelerated progression of HIV disease.^4^,^7^,^18^ This may be due to the transient reduction of HIV viral load during acute dengue infection.^5^ Unfortunately, there is a lack of large cohort study to validate these observations. To our knowledge, our study is the first matched case–control study to evaluate clinical and laboratory characteristics as well as outcome of DENV infection during first presentation and hospitalization, among patients with and without concomitant HIV infection.

DENV-HIV patients were more likely to present themselves significantly earlier to CDC for treatment, which suggested DENV-HIV patients are either feeling sicker, requiring earlier attention at a tertiary center, or they tend to have a more active health-seeking behavior. Because of the small sample size, diabetes and hypertension were not significantly different between the two groups, but these comorbidities might pose potential risk^19^ that warrant further investigation with an increasingly ageing HIV population and older DENV cases in many parts of the world.

At first presentation to CDC, there were no significant differences in the clinical symptoms and signs presented between DENV-HIV and DENV patients except for higher pulse rate in DENV-HIV patients. Over the course of hospitalization, DENV-HIV patients were less likely to have hemorrhagic manifestation and rash, compared with DENV patients, which supported the observations of previous published case series.^7^,^18^ Furthermore, this explained for the smaller proportion of DHF among DENV-HIV patients compared with DENV patients. On the contrary, coinfection of DENV and chikungunya were more likely to have joint pain, rash, and diarrhea, but less likely to have myalgia, vomiting, and abdominal pain.^20^ This potentially illustrated the presence of differences in pathophysiology between the two different coinfections, which would be interesting for future study.

Clinical fluid accumulation, hepatomegaly, and severe organ involvement were associated with DENV-HIV patients, albeit not statistically significant over the period of hospitalization. Hence, close monitoring of DENV-HIV patients for organ involvement is critical to reduce morbidity. Interestingly, we noted that even though most of these DENV-HIV patients had evidence of plasma leakage, only some DENV-HIV patients fulfilled the DHF classification because of the lack of hemorrhagic manifestation. Instead, there were more DENV-HIV patients classified as severe dengue based on the WHO 2009 dengue classification as these patients had fulfilled either severe plasma leakage or severe bleeding or severe organ involvement. This suggested that HIV might have altered DENV pathogenesis over time during hospitalization. However, further study is warranted to determine the mechanism involved in severe dengue progression in DENV-HIV patients.

DENV-HIV patients were found to have higher eosinophil proportion and pulse rate, but lower serum hematocrit level compared with DENV patients during first presentation and hospitalization. Eosinophilia is common in HIV-infected individuals and associated with parasitic infections,^21^ pruritic conditions,^22^ drug allergy,^23^ and Kaposi's sarcoma.^24^ Nevertheless, higher eosinophil counts were not significantly correlated with immune activation, altered HIV viral load,^25^,^26^ or thrombocytopenia and granulocytopenia in dengue hemorrhagic fever.^27^ Instead, the higher pulse rate and lower hematocrit among DENV-HIV cases were of concern. In an independent study comparing clinical and laboratory characteristics of DENV patients at first presentation and 24 hours prior to intensive care unit (ICU) admission, high pulse rate and lower hematocrit level were shown to be predictive factors of ICU admission compared with DENV patients that did not require ICU care.^28^ In another study in Singapore, it was shown that tachycardia on admission was independently associated with DENV mortality.^29^ Furthermore, adult DENV patients coinfected with bacterial infection had higher pulse rate and lower hematocrit,^30^ which was similarly observed in DENV-HIV patients in this study. Furthermore, lower serum hematocrit level may be due to DENV-HIV patients being on zidovudine, which is associated with anemia.^31^

Numerous reports suggested significant transient increase in HIV viral load when coinfected^32^ with malaria,^33^ leishmaniasis,^34^ Chagas disease,^35^ and herpes simplex virus.^36^ However, there were also reports of HIV suppression when coinfected with acute scrub typhus^37^ and measles.^38^ In addition, coinfection with GB virus type C (GBV-C), which belongs to the family Flaviviridae like DENV, was associated with improved survival among HIV-infected patients.^39^,^40^ This was hypothesized as a result of activation of HIV-inhibitory chemokines^41^ as well as the inhibitory role of GBV-C NS5A phosphoprotein against HIV replication in CD4+ T cells.^9^,^41^ Other studies also suggested DENV NS5 protein had a role in HIV-suppressing effect during acute infection.^8^,^9^ Hence, these observations may potentially explain the similar clinical manifestations and limited laboratory differences between DENV-HIV patients and DENV patients at first presentation and during hospitalization. However, DENV-HIV patients are likely to progress to severe outcome based on the WHO 2009 classification. Similar severe outcome trend is also observed during DENV coinfection with malaria.^42^–^44^ For future study, it would be interesting to evaluate HIV and DENV viral load prospectively over the course of DENV infection, to try to understand the impact of viral load and antiretroviral therapy on the disease outcome among DENV-HIV and DENV patients.

There are several limitations in this study. First, the small sample size of DENV-HIV cases might have limited statistical power to detect true associations, and associations had to be interpreted with caution. The small number of coinfected cases is likely due to the relatively low prevalence of HIV at 1,155 per million population (0.12%) in Singapore,^17^ as compared with neighboring countries in Asia.^3^ Hence, a matched case–control study design was used to maximize the efficiency of the small sample size. Unfortunately, because of the rigorous matching factors required, we do not have sufficient additional well-matched subjects from our cohort to repeat the analysis. Moreover, it is likely to introduce more biases if we use another random sample from the population of potential matches as these new controls are unlikely to represent the general dengue population with no HIV infection during the same period of recruitment due to other unknown confounding factors. Second, one dengue IgG positive coinfected patient was also included in the study because the patient fulfill the WHO probable dengue criteria, which has also been used and published widely. Furthermore, serology is one of the factors that was used in matching the controls and cases, and hence, it is unlikely to generate significant misclassification bias that will result in inconclusive inference. Third, there was a lack of HIV status of the controls, but the differential misclassification bias would be small as the prevalence of HIV is low, as highlighted above. Fourth, there was a lack of prospective daily HIV and DENV viral load assessment over the different phases of DENV infection to correlate with the clinical and laboratory characteristics. Fifth, the generalizability of these findings may be limited because only the Chinese DENV-HIV patients were found in the database. Different ethnics groups and environmental factors may also affect the overall findings, which would require future studies to investigate their impact. Next, the subjects involved in this study belong to a hospital cohort, where milder coinfected cases may be missed. Hence, the findings may not be generalizable in the community setting. Finally, the antiretroviral therapy for the DENV-HIV patients may have influenced the disease progression and clinical outcome, which could not be controlled in this study because of the small number of cases without drug therapy. As such, one should always keep these limitations into consideration when interpreting the results of this study.

In conclusion, we presented evidence to suggest DENV-HIV patients may be more likely to develop severe dengue outcome based on the WHO 2009 classification criteria, mainly due to severe plasma leakage. Clinicians should remain cautious when triaging DENV-HIV patients at first presentation, and close monitoring of these patients should be recommended as part of dengue clinical care and management. Future study with a larger number of DENV-HIV cases and a systematic meta-analysis would be required to validate these findings.

## Supplementary Material

Supplemental Tables.

## Figures and Tables

**Table 1:** DENV-HIV coinfected case listing

Patient	1	2	3	4	5	6	7	8	9	10
Age	59	40	67	33	32	72	57	39	47	47
Gender	Male	Male	Male	Male	Female	Male	Male	Male	Male	Male
Laboratory DENV diagnosis	PCR positive	IgM positive	IgM positive	IgM positive	IgG positive	IgM positive	PCR positive	IgM positive	PCR positive	IgM positive
WHO 1997 (outcome)	DHF	DF	DSS	DF	DHF	DF	DF	DF	DF	DF
Fever	Yes	Yes	Yes	Yes	Yes	Yes	Yes	Yes	Yes	Yes
Plasma leakage	Yes	No	Yes	Yes	Yes	Yes	Yes	No	Yes	No
Thrombocytopenia	Yes	Yes	Yes	Yes	Yes	Yes	Yes	Yes	Yes	Yes
Hemorrhagic manifestation	Yes	Yes	Yes	No	Yes	No	No	No	No	No
WHO 2009 (outcome)	SD	DF w/WS	SD	DF	SD	SD	DF w/WS	DF w/WS	SD	DF
Severe plasma leakage*	Yes	No	Yes	No	No	Yes	No	No	Yes	No
Severe bleeding	No	No	Yes	No	Yes	No	No	No	No	No
Severe organ involvement	No	No	No	No	No	Yes†	No	No	No	No
Comorbidities/disorders	Hypertension	None	Lung, renal	None	Diabetes, lung, renal	Asthma, cardiac, lung	None	None	Lung	None
Months post-HIV diagnosis at first dengue presentation	58	129	0	36	31	37	37	72	11	11
CD4 counts										
6 months before dengue	303	405	−	5	79‡	84	239	821	79	123
6 months after dengue	423	−	10	46	−	103	233	−	144	269
AIDS	Yes	No	Yes	Yes	Yes	Yes	Yes	No	Yes	No
HAART upon dengue diagnosis	Zidovudine, lamivudine, efavirenz	Nevirapine, ritonavir, saquinavir	No	Stavudine, lamivudine, nevirapine	Stavudine, lamivudine, nevirapine	No	Lopinavir, ritonavir	Lopinavir, ritonavir, lamivudine, zidovudine	Zidovudine, lamivudine, efavirenz	Zidovudine, lamvudine, efavirenz

AIDS = acquired immunodeficiency syndrome; DENV = dengue virus; DHF = dengue hemorrhagic fever; DF = dengue fever; DSS = dengue shock syndrome; HAART = highly active antiretroviral therapy; HIV = human immunodeficiency virus; PCR = polymerase chain reaction; w/ = with; WS = warning signs; SD = severe dengue.

* Plasma leakage and shock.^16^

† Impaired consciousness.

‡ Seven months before dengue.

**Table 2:** Severity characteristics of dengue patients with and without HIV infection

Variables	DENV-HIV cases (*N* = 10)	%	DENV controls (*N* = 40)	%	COR	*P* value	95% CI
WHO 1997 (at presentation)							
DF	10	100	28	70	−	−	−
DHF/DSS	0	0	12	30	−	−	−
WHO 2009 (at presentation)							
Non-severe dengue	9	90	34	85	1	−	−
Severe dengue	1	10	6	15	0.58	0.657	0.05–6.36
WHO 1997 (clinical outcome)							
DF	7	70	19	47.5	1	−	−
DHF/DSS	3	30	21	52.5	0.40	0.215	0.09–1.71
WHO 2009 (clinical outcome)							
Non-severe dengue	5	50	32	80	1	−	−
Severe dengue	5	50	8	20	**5.72**	**0.049**	**1.01**–**32.64**
Median DPP to DHF (range)	6 (2–7)	−	3 (2–4)	−	1.01	0.961	0.63–1.63
Median DPP to DSS (range)	6*	−	2.5 (2–3)	−	−	−	−
Median DPP to severe dengue (range)	2 (2–3)	−	2.5 (2–3)	−	2.21	0.069	0.94–5.18
Median DPF at presentation (IQR)	3 (2–4.8)	−	4 (4–5.3)	−	**0.47**	**0.032**	**0.24**–**0.94**
Median LOS in hospital (IQR)	10.5 (5.5–26.3)	−	5 (4–6)	−	1.86	0.054	0.99–3.50

DENV = dengue virus; DHF = dengue hemorrhagic fever; DF= dengue fever; DPF = days post fever onset; DPP = days post presentation; DSS = dengue shock syndrome; HIV = human immunodeficiency virus; CI = confidence interval; COR = conditional odds ratio; IQR = interquartile range; LOS = length of stay; WHO = World Health Organization. Numbers in bold highlight the estimated risk effect, *P*-value and 95% CI with significant statistical emphasis.

* 1 DSS.

**Table 3:** Key clinical characteristics of dengue patients with and without HIV infection

Variables	DENV-HIV cases (*N* = 10)	%	DENV controls (*N* = 40)	%	COR	*P* value	95% CI	ACOR	*P* value	95% CI
At presentation										
Pulse rate/minute (IQR)	93 (89.3–99)	−	75 (65–85)	−	**1.12**	**0.006**	**1.03**–**1.22**	**1.13**	**0.021**	**1.02**–**1.25**
During hospitalization										
Temperature (°C)	39.2 (38.3–39.9)	−	38.3 (37.5–38.9)	−	**2.83**	**0.02**	**1.18**–**6.79**	80.7	0.075	0.64–10,168
Pulse rate/minute (IQR)	111 (101.3–120.8)	−	90 (83.5–99)	−	**1.13**	**0.009**	**1.03**–**1.23**	**1.11**	**0.026**	**1.01**–**1.22**
Hemorrhagic manifestation										
Yes	4	40	32	80	**0.16**	**0.027**	**0.03**–**0.81**	0.13	0.054	0.02–1.03
Any rash										
Yes	3	30	33	82.5	**0.11**	**0.007**	**0.22**–**0.54**	**0.16**	**0.040**	**0.03**–**0.92**
Clinical fluid accumulation										
Yes	1	10	3	7.5	1.33	0.803	0.14–12.82	3.18	0.407	0.21–48.8
Hepatomegaly										
Yes	2	20	4	10	2.67	0.358	0.33–21.5	13.3	0.115	0.53–334
Hematocrit rise with rapid platelet drop										
Yes	4	40	8	20	1.95	0.455	0.34–11.23	1.56	0.655	0.22–10.97
Hypotension										
Yes	3	30	7	17.5	2.07	0.386	0.40–10.69	1.79	0.545	0.27–11.81
Narrow pulse pressure										
Yes	1	12.5	1	2.86	4	0.327	0.25–63.95	2.51	0.942	3.92e-11–1.61e1
Severe bleeding										
Yes	2	20	5	12.5	1.77	0.545	0.28–11.12	0.98	0.985	0.09–10.16
Severe organ involvement										
Yes	1	10	3	7.5	1.63	0.753	0.08–34.64	4.03	0.783	0.002–83,615

ACOR = adjusted conditional odds ratio; CI = confidence interval; COR = conditional odds ratio; DENV = dengue virus; HIV = human immunodeficiency virus; IQR = interquartile range.

ACOR adjusted by days post fever onset at first dengue presentation. Numbers in bold highlight the estimated risk effect, *P*-value and 95% CI with significant statistical emphasis.

**Table 4:** Significant laboratory characteristics of dengue patients with and without HIV infection

Variables (IQR)	DENV-HIV cases (*N* = 10)	DENV controls (*N* = 40)	COR	*P* value	95% CI	ACOR	*P* value	95% CI
At presentation								
White cell count (×10^9^/L)	3.85 (3.03–5.63)	2.8 (2–3.4)	**1.63**	**0.028**	**1.06**–**2.51**	1.42	0.15	0.88–2.29
Proportion of neutrophils (%)	72.15 (56.1–84.8)	58.5 (45.5–67.5)	**1.09**	**0.033**	**1.01**–**1.17**	1.06	0.182	0.97–1.16
Proportion of monocytes (%)	6.25 (4.6–10.6)	11.6 (9.3–15.6)	**0.81**	**0.043**	**0.66**–**0.99**	0.828	0.101	0.66–1.04
Proportion of eosinophils (%)	1 (0.4–2.2)	0.1 (0–0.4)	1.63	0.081	0.94–2.82	**3.07**	**0.029**	**1.12**–**8.41**
Hematocrit (%)	34.4 (17.7–40.7)	40.1 (24.4–48.8)	**0.78**	**0.012**	**0.64**–**0.95**	**0.79**	**0.035**	**0.64**–**0.98**
During hospitalization								
Proportion of neutrophils (%)	78.3 (62.3–88.4)	64.8 (58.8–71.2)	**1.07**	**0.046**	**1.00**–**1.15**	1.05	0.246	0.97–1.14
Proportion of eosinophil (%)	5.4 (4–6)	1.95 (1–3)	**1.72**	**0.022**	**1.08**–**2.72**	**1.90**	**0.010**	**1.17**–**3.09**
Hematocrit (%)	37.6 (35.9–46.4)	45.3 (42.9–48.7)	**0.73**	**0.015**	**0.56**–**0.94**	**0.74**	**0.043**	**0.56**–**0.99**
Serum potassium (mmol/L)	3.1 (2.9–3.3)	3.4 (3.2–3.7)	**0.03**	**0.015**	**0.002**–**0.49**	**0.01**	**0.041**	**0.001**–**0.81**
Serum albumin (g/L)	28 (22–35)	35 (32.3–38)	**0.83**	**0.033**	**0.69**–**0.98**	0.86	0.136	0.70–1.05

ACOR = adjusted conditional odds ratio; CI = confidence interval; COR = conditional odds ratio; DENV = dengue virus; HIV = human immunodeficiency virus; IQR = interquartile range.

ACOR adjusted by days post fever onset at first dengue presentation. Numbers in bold highlight the estimated risk effect, *P*-value and 95% CI with significant statistical emphasis.
