# Supplementary material for: Differential Clinical Outcome of Dengue Infection among Patients with and without HIV Infection: A Matched Case–Control Study
Source: Am J Trop Med Hyg. 2015 Jun 3;92(6):1156–62. doi: 10.4269/ajtmh.15-0031 (PMC4458819; doi:10.4269/ajtmh.15-0031)
Supplement: Supplementary file 1 [file SD4.pdf]

Supplemental Table 1

Demographic characteristics and preexisting conditions of dengue patients with and without HIV infection

| Variables | DENV-HIV cases ($N$ = 10) | % | DENV controls ($N$ = 40) | % | COR | $P$ value | 95% CI |
|---|---|---|---|---|---|---|---|
| Median age* (IQR) | 47 (39–58) | – | 44 (38–55) | – | 1.19 | 0.136 | 0.95–1.50 |
| Age groups | | | | | | | |
| 30–39 | 3 | 30 | 16 | 40 | – | – | – |
| 40–49 | 3 | 30 | 6 | 15 | – | – | – |
| 50–59 | 2 | 20 | 13 | 32.5 | – | – | – |
| ≥ 60 | 2 | 20 | 5 | 12.5 | – | – | – |
| Gender* | | | | | | | |
| Female | 1 | 10 | 4 | 10 | – | – | – |
| Laboratory diagnosis* | | | | | | | |
| PCR+ | 3 | 30 | 12 | 30 | – | – | – |
| Serology+ | 7 | 70 | 28 | 70 | – | – | – |
| IgG | | | | | | | |
| IgG+ | 9 | 90 | 34 | 85 | 1.72 | 0.657 | 0.16–18.75 |
| Year of dengue infection* | | | | | | | |
| 2005–2006 | 6 | 60 | 24 | 60 | – | – | – |
| 2007–2008 | 4 | 40 | 16 | 40 | – | – | – |
| Patient type* | | | | | | | |
| Inpatient | 9 | 90 | 36 | 90 | – | – | – |
| Outpatient | 1 | 10 | 4 | 10 | – | – | – |
| Diabetes mellitus | | | | | | | |
| Yes | 1 | 10 | 3 | 7.5 | 1.33 | 0.803 | 0.14–12.82 |
| Hypertension | | | | | | | |
| Yes | 1 | 10 | 8 | 20 | 0.428 | 0.46 | 0.045–4.05 |
| Hyperlipidemia | | | | | | | |
| Yes | 0 | 0 | 4 | 10 | – | – | – |
| Asthma | | | | | | | |
| Yes | 1 | 10 | 0 | 0 | – | – | – |
| Cardiac disorder | | | | | | | |
| Yes | 1 | 10 | 1 | 2.5 | – | – | – |
| Lung disorder | | | | | | | |
| Yes | 4 | 40 | 0 | 0 | – | – | – |
| Liver disorder | | | | | | | |
| Yes | 0 | 0 | 1 | 2.5 | – | – | – |
| Renal disorder | | | | | | | |
| Yes | 2 | 20 | 2 | 5 | 4 | 0.166 | 0.56–28.40 |
| Median HIV months prior to dengue (IQR) | 36.5 (16–52.75) | | NA | – | – | – | – |
| Median HIV viral load (IQR) | 50 (50–251) | | NA | – | – | – | – |
| Median CD4 count 6 months before dengue (IQR) | 123 (79–303) | | NA | – | – | – | – |
| Median CD4 count 6 months after dengue (IQR) | 144 (74.5–251) | | NA | – | – | – | – |
| Median lowest CD4 count recorded (IQR) | 45 (37–75.5) | | NA | – | – | – | – |
| AIDS | 7 | 70 | 0 | 0 | – | – | – |

AIDS = acquired immunodeficiency syndrome; CI = confidence interval; COR = conditional odds ratio; DENV = dengue virus; HIV = human immunodeficiency virus; IQR = interquartile range.

*Matched variables.

Supplemental Table 2

Clinical characteristics of dengue patients with and without HIV infection at presentation

| Variables | DENV-HIV cases ($N$ = 10) | % | DENV controls ($N$ = 40) | % | COR | *P* value | 95% CI | ACOR | *P* value | 95% CI |
|---|---|---|---|---|---|---|---|---|---|---|
| Hemorrhagic manifestation | | | | | | | | | | |
| Yes | 1 | 10 | 27 | 67.5 | 0.08 | 0.051 | 0.01–1.10 | 0.07 | 0.09 | 0.01–1.48 |
| Any rash | | | | | | | | | | |
| Yes | 1 | 10 | 19 | 47.5 | 0.12 | 0.053 | 0.01–1.03 | 0.11 | 0.100 | 0.01–1.54 |
| Leucopenia | | | | | | | | | | |
| Yes | 5 | 50 | 30 | 75 | 0.33 | 0.125 | 0.80–1.36 | 0.64 | 0.574 | 0.13–3.05 |
| Nausea/vomiting | | | | | | | | | | |
| Yes | 4 | 40 | 23 | 57.5 | 0.47 | 0.320 | 0.11–2.08 | 0.41 | 0.293 | 0.80–2.15 |
| Aches and pains | | | | | | | | | | |
| Yes | 5 | 50 | 32 | 80 | 0.26 | 0.073 | 0.06–1.14 | 0.34 | 0.18 | 0.07–1.64 |
| Any warning sign | | | | | | | | | | |
| Yes | 7 | 70 | 25 | 62.5 | 1.40 | 0.66 | 0.31–6.30 | 1 | 1 | 0.20–5.08 |
| Abdominal pain/tenderness | | | | | | | | | | |
| Yes | 2 | 20 | 7 | 17.5 | 1.16 | 0.86 | 0.22–6.23 | 1.12 | 0.92 | 0.11–11.21 |
| Persistent vomiting | | | | | | | | | | |
| Yes | 0 | 0 | 0 | 0 | – | – | – | – | – | – |
| Clinical fluid accumulation | | | | | | | | | | |
| Yes | 0 | 0 | 0 | 0 | – | – | – | – | – | – |
| Mucosal bleed | | | | | | | | | | |
| Yes | 1 | 10 | 10 | 25 | 0.33 | 0.323 | 0.04–2.96 | 0.313 | 0.30 | 0.035–2.84 |
| Lethargy | | | | | | | | | | |
| Yes | 4 | 40 | 9 | 22.5 | 2.24 | 0.278 | 0.52–9.57 | 2.4 | 0.28 | 0.50–11.67 |
| Hepatomegaly | | | | | | | | | | |
| Yes | 1 | 10 | 4 | 10 | 1 | 1 | 0.07–13.72 | 1.74 | 0.80 | 0.03–133.5 |
| Hematocrit rise with rapid platelet drop | | | | | | | | | | |
| Yes | 1 | 10 | 5 | 12.5 | 0.77 | 0.824 | 0.08–7.76 | 1.41 | 0.792 | 0.11–18.40 |
| Hypoproteinemia | | | | | | | | | | |
| Yes | 3 | 30 | 14 | 35 | 0.79 | 0.763 | 0.17–3.62 | 0.95 | 0.954 | 0.16–5.54 |
| Thrombocytopenia | | | | | | | | | | |
| Yes | 7 | 70 | 35 | 87.5 | 0.35 | 0.204 | 0.07–1.78 | 0.49 | 0.453 | 0.08–3.17 |
| Tachycardia | | | | | | | | | | |
| Yes | 3 | 30 | 8 | 20 | 1.89 | 0.47 | 0.34–10.65 | 1.32 | 0.801 | 0.15–11.58 |
| Hypotension | | | | | | | | | | |
| Yes | 2 | 20 | 5 | 12.5 | 1.77 | 0.545 | 0.28–11.12 | 1.20 | 0.868 | 0.11–13.95 |
| Mechanical ventilation | | | | | | | | | | |
| Yes | 0 | 0 | 0 | 0 | – | – | – | – | – | – |
| Respiratory distress | | | | | | | | | | |
| Yes | 0 | 0 | 0 | 0 | – | – | – | – | – | – |
| Severe bleeding | | | | | | | | | | |
| Yes | 0 | 0 | 2 | 5 | – | – | – | – | – | – |
| Severe organ involvement | | | | | | | | | | |
| Yes | 0 | 0 | 2 | 5 | – | – | – | – | – | – |

ACOR = adjusted conditional odds ratio; CI = confidence interval; COR = conditional odds ratio; DENV = dengue virus; HIV = human immunodeficiency virus.
ACOR adjusted by days post fever onset at first dengue presentation.

Supplemental Table 3

Clinical characteristics of dengue patients with and without HIV infection during hospitalization

| Variables | DENV-HIV cases ($N$ = 10) | % | DENV controls ($N$ = 40) | % | COR | $P$ value | 95% CI | ACOR | $P$ value | 95% CI |
|---|---|---|---|---|---|---|---|---|---|---|
| Leucopenia | | | | | | | | | | |
| Yes | 7 | 70 | 33 | 82.5 | 0.50 | 0.384 | 0.10–2.40 | 0.94 | 0.940 | 0.17–5.24 |
| Nausea/vomiting | | | | | | | | | | |
| Yes | 5 | 50 | 26 | 65 | 0.53 | 0.381 | 0.13–2.20 | 0.37 | 0.214 | 0.08–1.79 |
| Aches and pains | | | | | | | | | | |
| Yes | 8 | 80 | 34 | 85 | 0.72 | 0.707 | 0.13–4.08 | 0.53 | 0.546 | 0.07–4.23 |
| Any warning sign | | | | | | | | | | |
| Yes | 8 | 80 | 29 | 72.5 | 1.65 | 0.597 | 0.26–10.51 | 1.20 | 0.852 | 0.18–7.91 |
| Abdominal pain/tenderness | | | | | | | | | | |
| Yes | 4 | 40 | 12 | 30 | 1.56 | 0.549 | 0.37–6.66 | 1.09 | 0.933 | 0.15–7.68 |
| Persistent vomiting | | | | | | | | | | |
| Yes | 0 | 0 | 3 | 7.5 | – | – | – | – | – | – |
| Lethargy | | | | | | | | | | |
| Yes | 5 | 50 | 9 | 22.5 | 3.42 | 0.106 | 0.77–15.18 | 2.49 | 0.254 | 0.52–11.89 |
| Mucosal bleed | | | | | | | | | | |
| Yes | 1 | 10 | 16 | 40 | 0.17 | 0.110 | 0.02–1.48 | 0.18 | 0.133 | 0.02–1.69 |
| Hypoproteinemia | | | | | | | | | | |
| Yes | 5 | 55.6 | 18 | 54.6 | 0.73 | 0.689 | 0.15–3.44 | 0.71 | 0.70 | 0.13–3.91 |
| Thrombocytopenia | | | | | | | | | | |
| Yes | 10 | 100 | 40 | 100 | – | – | – | – | – | – |
| Tachycardia | | | | | | | | | | |
| Yes | 7 | 70 | 8 | 20 | – | – | – | – | – | – |
| Respiratory distress | | | | | | | | | | |
| Yes | 1 | 10 | 2 | 5 | 2 | 0.571 | 0.18–22.06 | 3.83 | 0.325 | 0.26–55.77 |
| Mechanical ventilation | | | | | | | | | | |
| Yes | 0 | 0 | 0 | 0 | – | – | – | – | – | – |

ACOR = adjusted conditional odds ratio; CI = confidence interval; COR = conditional odds ratio; DENV = dengue virus; HIV = human immunodeficiency virus.
ACOR adjusted by days post fever onset at first dengue presentation.

Supplemental Table 4

Laboratory characteristics of dengue patients with and without HIV infection at presentation

| Variable (IQR) | DENV-HIV cases ($N$ = 10) | DENV controls ($N$ = 40) | COR | $P$ value | 95% CI | ACOR | $P$ value | 95% CI |
|---|---|---|---|---|---|---|---|---|
| Temperature (°C) | 37.7 (37.4–38.4) | 37.8 (37–38.6) | 0.95 | 0.919 | 0.38–2.37 | 0.85 | 0.766 | 0.30–2.42 |
| Respiratory rate/minute | 19 (18–20) | 20 (18–20) | 0.49 | 0.128 | 0.20–1.23 | 0.22 | 0.215 | 0.02–2.38 |
| Oxygen saturation (%) | 99 (98–99.5) | 97.5 (96–98.3) | 1.97 | 0.281 | 0.57–6.76 | – | – | – |
| Proportion of lymphocytes (%) | 17.2 (8.4–29.3) | 21.6 (17.4–28.7) | 0.96 | 0.338 | 0.87–1.05 | 1.01 | 0.892 | 0.91–1.12 |
| Proportion of basophils (%) | 0.3 (0.2–0.5) | 0.1 (0–0.5) | 1.86 | 0.186 | 0.74–4.68 | 1.83 | 0.198 | 0.73–4.62 |
| Platelet ($10^9$/L) | 71.5 (44.8–111) | 60 (33–73.5) | 1.01 | 0.093 | 0.99–1.03 | 1.02 | 0.211 | 0.99–1.04 |
| Serum urea (mmol/L) | 5.9 (3.7–11.6) | 3.7 (2.7–4.7) | 1.50 | 0.077 | 0.96–2.34 | 1.38 | 0.321 | 0.73–2.58 |
| Serum creatinine (mmol/L) | 98 (97–101.5) | 100.5 (98–105) | 0.83 | 0.193 | 0.63–1.10 | 0.80 | 0.185 | 0.58–1.11 |
| Serum bilirubin | 13 (9–16) | 13 (10.3–15.8) | 1.05 | 0.409 | 0.93–1.18 | 1.04 | 0.597 | 0.89–1.22 |
| Serum AST (U/L) | 35 (22–158) | 89 (54–189.5) | 1 | 0.663 | 0.99–1.00 | 1 | 0.965 | 0.99–1.00 |
| Serum ALT (U/L) | 42 (20–127) | 60 (42–99.5) | 0.99 | 0.620 | 0.99–1.01 | 1 | 0.949 | 0.99–1.01 |
| Serum protein (g/L) | 63.5 (60.8–69.3) | 63 (59–70) | 0.97 | 0.325 | 0.91–1.03 | 0.98 | 0.466 | 0.91–1.04 |
| Serum albumin (g/L) | 33 (27–35) | 36 (35–39) | 0.87 | 0.118 | 0.73–1.04 | 0.93 | 0.461 | 0.76–1.13 |

ACOR = adjusted conditional odds ratio; ALT = alanine aminotransferase; AST = aspartate aminotransferase; CI = confidence interval; COR = conditional odds ratio; DENV = dengue virus; HIV = human immunodeficiency virus; IQR = interquartile range.
ACOR adjusted by days post fever onset at first dengue presentation.

SUPPLEMENTAL TABLE 5

Laboratory characteristics of DENV patients with and without HIV infection during hospitalization

| Variables (IQR) | DENV-HIV cases ($N$ = 10) | DENV controls ($N$ = 40) | COR | $P$ value | 95% CI | ACOR | $P$ value | 95% CI |
|---|---|---|---|---|---|---|---|---|
| Respiratory rate/minute | 18 (18–20) | 18 (16–18) | 1.25 | 0.256 | 0.85–1.86 | 1.25 | 0.318 | 0.80–1.95 |
| Oxygen saturation (%) | 95 (94–96) | 97.5 (96–98) | 0.66 | 0.054 | 0.43–1.01 | 0.72 | 0.133 | 0.46–1.11 |
| White cell count ($10^9$/L) | 3 (1.7–4.0) | 2.3 (1.9–3) | 1.31 | 0.224 | 0.85–2.01 | 1.13 | 0.624 | 0.69–1.85 |
| Proportion of lymphocytes (%) | 33.8 (20.9–44.2) | 38.8 (31.6–49.4) | 0.95 | 0.112 | 0.90–1.01 | 0.98 | 0.441 | 0.92–1.04 |
| Proportion of monocytes (%) | 14 (10.1–16) | 17.9 (14–24.8) | 0.86 | 0.051 | 0.73–1.00 | 0.89 | 0.191 | 0.75–1.06 |
| Proportion of basophils (%) | 0.7 (0.5–1.7) | 1.35 (1–2.6) | 0.65 | 0.159 | 0.36–1.18 | 0.68 | 0.225 | 0.37–1.26 |
| Platelet ($10^9$/L) | 33 (18–73.3) | 19.5 (10–45.5) | 1.03 | 0.080 | 0.99–1.06 | 1.02 | 0.251 | 0.98–1.07 |
| Serum urea (mmol/L) | 5.9 (3.7–12) | 3.8 (2.7–4.8) | 1.09 | 0.117 | 0.98–1.22 | 1.06 | 0.330 | 0.94–1.20 |
| Serum creatinine (μmol/L) | 103 (100–110) | 103 (98.3–105.8) | 1.03 | 0.691 | 0.88–1.22 | 1.03 | 0.716 | 0.86–1.24 |
| Serum bilirubin (μmol/L) | 13 (9–16) | 14 (11–20) | 1.05 | 0.377 | 0.94–1.18 | 1.02 | 0.783 | 0.89–1.17 |
| Serum AST (U/L) | 58.5 (25.3–135) | 118.5 (67–223.5) | 1 | 0.297 | 0.99–1.00 | 1 | 0.39 | 0.99–1.00 |
| Serum ALT (U/L) | 51.5 (21.3–119.8) | 71.5 (47.5– 153.3) | 0.99 | 0.304 | 0.98–1.01 | 0.99 | 0.333 | 0.98–1.01 |
| Serum protein (g/L) | 60 (46–69) | 62 (56–69) | 0.95 | 0.175 | 0.87–1.03 | 0.96 | 0.339 | 0.88–1.04 |

ACOR = adjusted conditional odds ratio; ALT = alanine aminotransferase; AST = aspartate aminotransferase; CI = confidence interval; COR = conditional odds ratio; DENV = dengue virus; HIV = human immunodeficiency virus; IQR = interquartile range.

ACOR adjusted by days post fever onset at first dengue presentation.
